# Examining intra- and inter-device reliability of pressure-mediated reflection spectroscopy in a multi-state sample of healthy adults

**DOI:** 10.1017/S136898002510116X

**Published:** 2025-09-25

**Authors:** Susan B. Sisson, Emily Helms, Shanon Casperson, Saima Hasnin, Stephanie Jilcott Pitts, Virginia C. Stage, Christopher R. Long, Taren Massey-Swindle, Dipti A. Dev, Ashlea Braun, Jodi D. Stookey, Rowena Cape, Jonathan Baldwin

**Affiliations:** 1 Department of Nutrition Sciences, University of Oklahoma Health Sciences Center, Oklahoma City, OK, USA; 2 USDA Agricultural Research Services, Grand Forks Human Nutrition Research Center, Grand Forks, ND, USA; 3 Department of Food Science and Human Nutrition, College of Agricultural, Consumer and Environmental Sciences, University of Illinois Urbana-Champaign, Urbana, IL, USA; 4 Department of Public Health, East Carolina University, Greenville, NC, USA; 5 Department of Agricultural and Human Sciences, NC State University, Raleigh, NC, USA; 6 Center for Nutrition and Health Impact, Omaha, NE, USA; 7 Department of Pediatrics, University of Arkansas for Medical Sciences, Little Rock, AR; Arkansas Children’s Research Institute, Little Rock, AR; Arkansas Children’s Nutrition Center, Little Rock, AR, USA; 8 Department of Child, Youth and Family Studies, College of Education and Human Sciences, University of Nebraska-Lincoln, Lincoln, NE, USA; 9 Department of Nutritional Sciences, College of Education & Human Sciences, Oklahoma State University, Stillwater, OK, USA; 10 TSET Health Promotion Research Center, Stephenson Cancer Center, University of Oklahoma Health Sciences, Tulsa, OK, USA; 11 Maternal, Child & Adolescent Health, San Francisco Department of Public Health, San Francisco, CA 94103, USA; 12 College of Allied Health, University of Oklahoma Health Sciences, Oklahoma City, OK, USA

**Keywords:** Veggie Meter, Dietary assessment, Biological assessment, Skin carotenoid

## Abstract

**Objective:**

To examine the intra- and inter-device reliability of devices using pressure-mediated reflection spectroscopy (the Veggie Meter®).

**Design:**

A cross-sectional research study was conducted across eight sites in the USA. Using two Veggie Meters® at each site, participants completed five, counter-balanced pairs of finger scans. Intra-device comparisons included intra-class correlation coefficients (ICC) and calculation of the CV and 95 % CI of each device/site; hypothesised to be ≤ 6 %. Inter-device comparisons included ICC, absolute relative differences (ARD) and 95 % CI, and equivalence; both hypothesised to be ≤ 10 %.

**Setting:**

Eight sites across the USA.

**Participants:**

Across sites, participants’ (*n* 282) average age ranged 24·7–39·0 years; sex ranged 60·0–85·7 % women and Non-Hispanic White ranged 20·0–94·3 %.

**Results:**

Intra-device ICC ranged from 0·77 to 0·99. The CV ranged from 6·2 to 14·2 %, with an average of 8·8 %. A majority (63 %; *n* 10) of the Veggie Meter® devices had significantly higher CV from the hypothesised 6 %. Inter-device ICC ranged from 0·58 to 0·94. The ARD ranged from 7·5 to 22·0 %, with an average of 13·9 %. ARD in a majority (*n* 5) of sites was significantly higher than the hypothesised 10 %. Five sites (63 %) demonstrated equivalence below the hypothesised 10 %.

**Conclusions:**

Our study demonstrates the intra-device and inter-device reliability to be moderate to high, as per ICC. The observed margin of difference within a device was up to 14 %, with an average of 9 %. The observed margin of difference between devices was up to 22 %, with an average of 14 % between devices.

Accurate and non-invasive assessment of fruit and vegetable intake is needed to overcome challenges associated with self-report measures. Technologies that measure skin carotenoids have emerged as a valid non-invasive method of assessing fruit and vegetable intake using resonance Raman spectroscopy and reflection spectroscopy (RS). The latter, RS, is relatively lower cost, portable and provides a rapid (less than 1 min) assessment of skin carotenoids. An RS device that is frequently used in recent public health nutrition studies is the Veggie Meter®^(1–8)^.

There is extremely high construct validity between the Veggie Meter®-assessed skin carotenoid score (SCS) and the score derived from the earlier established resonance Raman spectroscopy (*r* = 0·86–0·94)^(9,10)^. Veggie Meter®-assessed SCS in adults is highly correlated with total serum carotenoids (*r* = 0·81, *P* < 0·001)^(9)^ and total plasma carotenoids (*r* = 0·71, *P* < 0·001)^(10,11)^ and is sensitive to changes in dietary intake of carotenoids over relatively short time frames (e.g. 13·1 mg consumed daily for 3 weeks)^(9,12,13)^. Veggie Meter®-assessed SCS is also significantly associated with self-reported dietary recall in adults and children using a variety of recalls representing varied recall time frames^(11,14–19)^. Veggie Meter®-assessed SCS has high stability with sd of sequential measurements ranging between 3·4 % and 4·1 %^(9)^.

A better understanding of the reliability of the Veggie Meter® is needed considering its use in a variety of public health nutrition research and community-based settings. It is important to note that reliability needs to be examined in two manners: (1) intra-device reliability, or repeatability, as the similarity in SCS on the same individual, on the same day, on the same device and (2) inter-device reliability as the similarity in SCS on the same individual, on the same day and on a different device. The intra-device reliability of the Veggie Meter® has been examined in only two studies^(18,20)^. The CV ranged from 4·8 % to 9·1 % in these studies^(18,20)^. Inter-device reliability has been examined in only one study with a high reported intraclass correlation coefficient (ICC = 0·99)^(20)^. Further, the mean actual differences between devices ranged from 24·9 to 44·6 indicating 7·4–11·8 % difference between devices^(20)^. All participants were of similar ancestry and in a single geographic location limiting generalisability.

What is unknown is if multiple Veggie Meter® devices will produce similar SCS within the same person and how these scores can be compared between devices in geographically diverse populations. Understanding the equivalence between devices is an important aspect of inter-device reliability and can have research and practice implications (e.g. in cases where a research team has multiple Veggie Meter® devices and measures individuals longitudinally using either device). Therefore, the purpose of this study was to examine the intra-device reliability and inter-device reliability of Veggie Meter® devices in a sample of adults at eight sites across the USA. We hypothesised that the intra-device and inter-device ICC will be high (above 0·75). We expected the CV in SCS from multiple scans of the same participant on the same device would be <6 %^(18,20)^. We anticipated the absolute relative difference (ARD) in SCS from multiple Veggie Meter® devices would be within 10 %^(20)^. Further equivalence in SCS of the two devices was hypothesised to be 10 % or less.

## Methods

### Study design

This cross-sectional research study included a target of thirty-five participants per site across eight sites in the USA: University of Oklahoma Health Sciences in Oklahoma City, OK (coordinating site; OUHSC); University of Nebraska in Lincoln, Nebraska (UNL); East Carolina University and North Carolina State University collaborative, North Carolina (ECU/NC State); University of Arkansas for Medical Sciences (UAMS) campuses at Fayetteville (UAMS – Fayetteville) and Little Rock (UAMS – Little Rock); Oklahoma State University in Stillwater, OK (OSU); the Grand Forks Human Nutrition Research Center in Grand Forks, North Dakota (GFHNRC) and the San Francisco Department of Public Health (SFDPH). All sites participated in protocol development, study design and data collection. OUHSC was the coordinating site and developed the online survey data collection tools, housed de-identified data and conducted data analyses. Sample size was determined based on power to determine a 5 % absolute magnitude of difference within devices (*n* 21) and between devices (*n* 29). The absolute magnitude of difference between scores within a participant for our actual pilot data of 4 people was 9·1 %. These pilot data suffer from a very wide CI with such a small sample. If the actual difference was modified to 6·5 %, as would be more likely with a larger dataset, twenty-one participants would be needed. The absolute magnitude of difference between devices in our pilot data was 9·6 %. If the actual difference was modified to 7 %, as would be more likely with a larger dataset, twenty-nine participants would be needed. Enrollment was increased to account for variability and missing data.

Following consent, participants completed a questionnaire and surveys on demographic characteristics, skin light reactivity^(21)^ and a carotenoid-rich food source screener^(12)^. Using two Veggie Meters® at each site, participants completed five pairs of skin carotenoid scans using the single scan function for a total of ten scans of their non-dominant ring finger^(22)^. Each University’s Institutional Review Board approved each site’s study (OUHSC #14488; UNL #20221222110EP; ECU #22-001293; UAMS #274841; OSU #IRB-22-449-STW; GFNHRC reviewed by University of North Dakota #0005363; SFDPH #20-30320). Following participation, some participants received compensation of varying ($0, $15, $25 and $35) amounts based on funding availability and site-level IRB approval. Data collection occurred from July 2022 through November 2023.

### Recruitment/Participants

Participants (*n* 282) were recruited to research laboratories at each site (OUHSC *n* 35; UNL *n* 35; ECU *n* 35; UAMS-Fayetteville *n* 33; UAMS-Little Rock *n* 37; OSU *n* 37; GFHNRC *n* 35 and SFDPH *n* 35). Participants were English-speaking, healthy adults 18–65 years of age and had no impairments or deformities to his/her non-dominant ring finger. Participants were recruited by convenience, word of mouth and email. Due to possible skin discolouration, participants who had eaten Doritos, Cheetos or Takis in the 3 days prior to their appointment or who used self-tanning lotion in the 2 weeks prior to their scheduled Veggie Meter® appointment were rescheduled or excluded from the study if they did not wish to reschedule.

Interested participants were screened for eligibility and scheduled until recruitment goals were met. Eligibility exclusions included finger scarring (OUHSC *n* 1) and using self-tanning lotion (UNL *n* 1).

### Procedures

Veggie Meter® equipment descriptions and any protocol variations across sites are described in online supplementary material, Supplemental Table 1. Prior to participant arrival, Veggie Meters® were calibrated using the factory recommended procedures. Veggie Meters® are re-calibrated every 60 min of data collection at all sites except UNL in which they are calibrated every 30 min. Veggie Meters® are identified within each laboratory as device 1 or 2 to ensure counterbalancing of finger scans.

Upon arrival at the research laboratory, participants provided informed consent. Height and weight were measured at only five of the sites. Anthropometric measures were excluded at UAMS Fayetteville due to privacy concerns as many participants were faculty and staff in the department during data collection and self-reported at two sites for cultural preferences (SFDPH) and privacy (UAMS Little Rock).

For the finger scans, participants inserted their non-dominant ring finger into one Veggie Meter®. Upon completion of the first scan, participants inserted the same finger into the other Veggie Meter® to complete the first pair of scans. Each scan took less than a minute to produce an SCS. For each pair of scans, the order of the devices was counterbalanced. For example, for the first pair of scans, the participant was scanned on device 1 then device 2 (arbitrarily assigned at each site but remained consistent throughout the study); for the second pair of scans, the participant was scanned on device 2 then device 1. Participants were alternately assigned to start scan 1 of the first pair of scans on either device 1 or 2. For example, participant 1 started their first scan with device 1; participant 2 started their first scan with device 2, and so on. Participants rested 5 minutes between pairs of scans to allow for full blood flow to return to the finger. During the 5-minute breaks between scan sets, participants completed questionnaires. Thus, each participant had a total of five pairs of SCS representing a total of ten SCS, five scores from device 1 and 5 scores from device 2.

Most sites collected data on a single research participant at a time; however, two sites (UAMS-Little Rock and SFPHD) collected data on two participants simultaneously and switched participants back and forth across both devices. For those sites collecting data on a single participant, the participants washed hands prior to starting scans, and the device was wiped with alcohol prior to scans. For those sites collecting data on multiple participants simultaneously, the device was wiped with alcohol and allowed to dry between each participant’s finger scan.

Spurious values were occasionally observed and handled differently across sites. All but two sites recorded each value as displayed by the equipment. However, two sites used different protocols. When values were observed greater than 100 points higher or lower than the immediately preceding score, GFNHRC would remove the participant’s finger, reposition and rescan. Spurious values were attributed to incorrect finger placement on the Veggie Meter® lens. At UNL, when values >10 % different from the previous scan were observed, the device was recalibrated and the participant rescanned.

### Measures

#### Veggie Meter®

The Veggie Meter® device is a tool used to measure skin carotenoids using pressure-mediated RS. The spectroscopy-based device utilise white LED light and reflection optical signaling^(9,23)^. Slight pressure (∼1 atm) temporarily moves blood from the fingertip for optical assessment of the reflected light to be identified by the spectrograph^(9,23)^.

Veggie Meters® used across sites varied by the date of purchase. While manufacturers indicated no change in the equipment or anticipated scores over time, investigators observed minor nuances in the instructional manuals regarding warm up time and calibration reference numbers (see online supplementary material, Supplemental Table 1). Further, Obana 2023^(20)^ indicated variation in SCS with devices purchased over different time frames.

#### Anthropometric measures

For those sites measuring height (cm) and weight (kg), each participant’s anthropometric measures were collected in bare or socked feet. Height was measured to the nearest half centimeter. Weight was recorded without heavy clothes and to the nearest hundredth of a kilogram. Tools used to collect height and weight varied by site and described in online supplementary material, Supplemental Table 2. The questionnaire obtained participant information regarding sex, education, marital status, race/ethnicity, age, smoking/vaping status and Fitzpatrick skin reactivity^(21,24)^.

### Statistical analysis

Sites were analysed separately since examination of these intra-individual and inter-individual variability will require the same individual being scanned on each device. Further, slight deviations in the protocol around spurious values may introduce lower intra-device and inter-device variability. Demographic characteristics were calculated for participants at each site. To fully examine intra- and inter-device reliability, multiple analyses were conducted and are described herein. Mathematical calculations, respectively, described below, were used to quantify the magnitude of the variance within and between devices. SAS version 9·4 (SAS Institutes) was used for all calculations.

#### Intra-device comparisons

ICC are a statistic that describes how strongly units within two groups resemble each other and is often used as a measure of intra- and inter-rater agreement^(25,26)^. A higher ICC indicates higher agreement across devices or raters; an ICC of <0·5 is poor, 0·5–0·75 is moderate, 0·75–0·9 is good and above 0·9 is excellent^(25)^. An ICC ≥ 0·75 was hypothesised. The 95 % CI describe the range (i.e. variance) around the point estimate (ICC, in this case). ICC and 95 % CI were calculated across all five scores within each device for each participant to determine the reliability of scores within an individual.

CV (CV = sd/mean) is a measure of the precision of a sample mean (i.e. data dispersion). CV presents the standard deviation (sd) as a percent of the mean and is calculated by CV = sd/mean. CV for each device were calculated to determine the relative measure of variability within an individual’s five scores, yielding two CV for each participant, one for each Veggie Meter® device. CV for each device (Veggie Meter® 1 and Veggie Meter® 2) were calculated from averaging all respective device CV across all site participants. CV across each site were averaged. A 95 % CI was calculated to determine if the variation of each site’s CV differs from the hypothesised 6 %, based on previous studies^(18,20)^. If the upper and lower values of the 95 % CI do not cross the hypothesised value, then it can be determined that the CV for that device is significantly lower or higher than the hypothesised value. A bootstrapped approach was used to determine the 95 % CI since the CV were not normally distributed. Since analysis involved separate computations for each clinical site, normality was assessed for data from each of the eight sites. Six (ECU/NC State, UAMS-Fayetteville, UAMS-Little Rock, OSU, GFNHRC and SFDPH) site’s CV were not normally distributed. In the case of a normally distributed CV, that site’s 95 % CI was computed using parametric methods; however, if the site’s CV was not normally distributed, a bootstrap procedure (5000 bootstrap samples) was used to obtain 95 % CI from the 2·5th percentile and 97·5th percentile of the bootstrap distribution. We chose to use both ICC and CV because they describe intra-device reliability differently: the ICC estimates intra-device variability as a fraction of the total variability, and CV measures intra-device variability as a fraction of the mean measure.

#### Inter-device comparisons

To determine the level of agreement between devices at each respective site, ICC were calculated across each pair of SCS taken from both devices (Veggie Meter® 1 and Veggie Meter® 2) at a given site.

The ARD of the SCS from device 1 and device 2 was determined relative to the mean of the two scores in the pair for each of the five pairs (│device 2 – device 1│/mean of device 1 and device 2). The participant’s mean relative difference was determined by averaging the relative difference for each of the five pairs of scans. The 95 % CI describe the range (i.e. variance) around the point estimate (ARD, in this case). If the upper and lower values of the 95 % CI do not cross the hypothesised value, then it can be determined that the ARD is significantly lower or higher than the hypothesised value. A 95 % CI was calculated to determine if the variation of each site’s ARD differs from the hypothesised 10 %^(20)^. Much like CV above, six site’s (UNL, ECU/NC State, UAMS-Fayetteville, UAMS-Little Rock, OSU, GFNHRC) ARD was not normally distributed. If normally distributed, the 95 % CI was computed using parametric methods, and if non-normally distributed then a 5000-sample bootstrap procedure was used to compute the 95 % CI.

Skin carotenoid score equivalence between the two devices was determined using CI intervals on the percent deviation, relative to their mean ([Device 1 – Device 2]/[mean of Devices 1 and 2] × 100), of the two devices per site. Equivalence was examined at 10 %.

## Results

Descriptive characteristics of participants enrolled at each site are included in Table 1. Across all eight sites, the participants were mostly young adults (average 31 years), female, Non-Hispanic White, had college or graduate/professional degrees, non-smokers and about half of the sample was classified as overweight/obese.

**Table 1:** Mean, sd, frequency of participant descriptive characteristics across 8 sites (2022–2023)

	OUHSC (*n* 35)		UNL (*n* 35)		ECU/NC State (*n* 35)		UAMS-Fayetteville (*n* 33)		UAMS – Little Rock (*n* 37)		OSU (*n* 37)		GFNHRC (*n* 35)		SFDPH (*n* 35)	
	Mean	sd	Mean	sd	Mean	sd	Mean	sd	Mean	sd	Mean	sd	Mean	sd	Mean	sd
Age (years)	30·7	10·7	28·9	4·8	28·6	8·1	34·2	9·7	39	10·6	24·7	6·6	34·3	9·7	32·7	12·5
	%	*n*	%	*n*	%	*n*	%	*n*	%	*n*	%	*n*	%	*n*	%	*n*
Sex																
Male	22·9 %	8	40·0 %	14	31·4 %	11	36·4 %	12	16·7 %	6	37·1 %	13	22·9 %	8	14·3 %	5
Female	77·1 %	27	60·0 %	21	68·6 %	24	63·6 %	21	83·3 %	30	62·9 %	22	77·1 %	27	85·7 %	30
Race																
Hispanic American Indian	0 %	0	0 %	0	0 %	0	0 %	0	0 %	0	0 %	0	0 %	0	2·9 %	1
Hispanic Asian	0 %	0	0 %	0	0 %	0	0 %	0	2·8 %	1	0 %	0	0 %	0	2·9 %	1
Hispanic Other	0 %	0	0 %	0	2·9 %	1	3·0 %	1	0 %	0	0 %	0	0 %	0	0 %	0
Hispanic Black	2·9 %	1	0 %	0	0 %	0	0 %	0	0 %	0	0 %	0	0 %	0	0 %	0
Hispanic White	2·9 %	1	2·9 %	2	0 %	0	12·1 %	4	11·1 %	4	5·7 %	2	0 %	0	8·6 %	3
NH Black	5·7 %	2	5·7 %	2	20·0 %	7	3·0 %	1	30·6 %	11	0 %	0	0 %	0	11·4 %	4
NH Asian	14·3 %	5	71·4 %	25	8·6 %	3	3·0 %	1	5·6 %	2	11·4 %	4	0 %	0	31·4 %	11
NH White	71·4 %	25	20·0 %	7	68·6 %	24	69·7 %	23	50·0 %	18	77·1 %	27	94·3 %	33	40·0 %	14
NH American Indian	0 %	0	0 %	0	0 %	0	3·0 %	1	0 %	0	0 %	0	5·71 %	2	0 %	0
NH Pacific Islander	0 %	0	0 %	0	0 %	0	3·0 %	1	0 %	0	0 %	0	0 %	0	0 %	0
NH Other	0 %	0	0 %	0	0 %	0	0 %	0	0 %	0	2·9 %	1	0 %	0	0 %	0
No answer/ Don’t know	2·9 %	1	0 %	0	0 %	0	3·0 %	1	0 %	0	2·9 %	1	0 %	0	2·9 %	1
Level of education																
High School Grad/GED	0 %	0	0 %	0	11·4 %	4	3·0 %	1	5·6 %	2	8·6 %	3	2·9 %	1	5·7 %	2
Some College	5·7 %	2	8·6 %	3	11·4 %	4	9·1 %	3	8·3 %	3	34·3 %	12	25·7 %	9	5·7 %	2
College Grad	68·6 %	24	25·7 %	9	28·6 %	10	48·5 %	16	36·1 %	13	34·3 %	12	45·7 %	16	45·7 %	16
Prof/Grad School	25·7 %	9	65·7 %	23	48·6 %	17	39·4 %	13	50 %	18	22·9 %	8	25·7 %	9	42·9 %	15
Marital status																
Single	45·7 %	16	48·6 %	17	60 %	21	21·2 %	7	27·8 %	10	68·6 %	24	31·4 %	11	0 %	0
Living with partner	11·4 %	4	2·9 %	1	17·1 %	6	24·2 %	8	8·3 %	3	2·9 %	1	11·4 %	4	0 %	0
Married	37·1 %	13	48·6 %	17	22·9 %	8	45·5 %	15	58·3 %	21	22·9 %	8	54·3 %	19	0 %	0
Separated	0 %	0	0 %	0	0 %	0	3·0 %	1	5·6 %	2	2·9 %	1	0 %	0	0 %	0
Divorced	5·7 %	2	0 %	0	0 %	0	6·0 %	2	0 %	0	2·9 %	1	0 %	0	0 %	0
Prefer not to answer	0 %	0	0 %	0	0 %	0	0 %	0	0 %	0	0 %	0	2·9 %	1	100 %	35
Smoked 100 cigarettes in lifetime																
Yes	20 %	7	20 %	7	5·7 %	2	21·2 %	7	22·9 %	8	5·7 %	2	22·9 %	8	5·7 %	2
No	80 %	28	77·1 %	27	94·3 %	33	75·8 %	25	77·1 %	27	94·3 %	33	77·1 %	27	91·4 %	32
Don’t know/Not sure	0 %	0	2·7 %	1	0 %	0	3· %	1	0 %	0	0 %	0	0 %	0	2·9 %	1
BMI	26·3	5·7	26·6	6·3	27·1	5·6	n/a		28	6·9	25	4·9	30·2	7·1	24·5	4·4
BMI classification							n/a									
Underweight	2·9 %	1	8·6 %	3	5·7 %	2			5·4 %	2	2·7 %	1	2·9 %	1	2·9 %	1
Normal weight	51·4 %	18	34·3 %	12	40·0 %	14			29·7 %	11	59·5	22	17·1 %	6	51·4 %	18
Overweight	22·9 %	8	31·43 %	11	20·0 %	7			24·3 %	9	24·3 %	9	37·1 %	13	34·3 %	12
Obese	22·9 %	8	25·71 %	9	34·3 %	12			40·5 %	15	13·5 %	5	42·9 %	15	11·4 %	4
Skin reactivity score	19·1	5	21·3	4	20·4	5·3	17·7	5·5	19·5	6·5	16·7	6·3	16·6	6·3	20·6	6·3
Skin reactivity category																
Type I	0 %	0	0 %	0	2·9 %	1	6·1 %	2	5·4 %	2	10·8 %	4	14·3 %	5	2·9 %	1
Type II	14·3 %	5	0 %	0	8·6 %	3	12·1 %	4	21·6 %	8	24·3 %	9	22·9 %	8	14·3 %	5
Type III	45·7 %	16	37·1 %	13	37·1 %	13	54·6 %	18	29·7 %	11	40·5 %	15	42·9 %	15	28·6 %	10
Type IV	40 %	14	51·4 %	18	45·7 %	16	27·3 %	9	29·7 %	11	21·6 %	8	11·4 %	4	42·9 %	15
Type V	0 %	0	11·4 %	4	5·7 %	2	0 %	0	13·5 %	5	2·7 %	1	8·6 %	3	11·43 %	4
Type VI	0 %	0	0 %	0	0 %	0	0 %	0	0 %	0	0 %	0	0 %	0	0 %	0

OUHSC, Oklahoma Health Sciences in Oklahoma City; UNL, University of Nebraska in Lincoln, Nebraska; ECU, East Carolina University; NC, North Carolina; UAMS, University of Arkansas for Medical Sciences; OSU, Oklahoma State University; SFDPH, San Francisco Department of Public Health.

**Figure 1:**
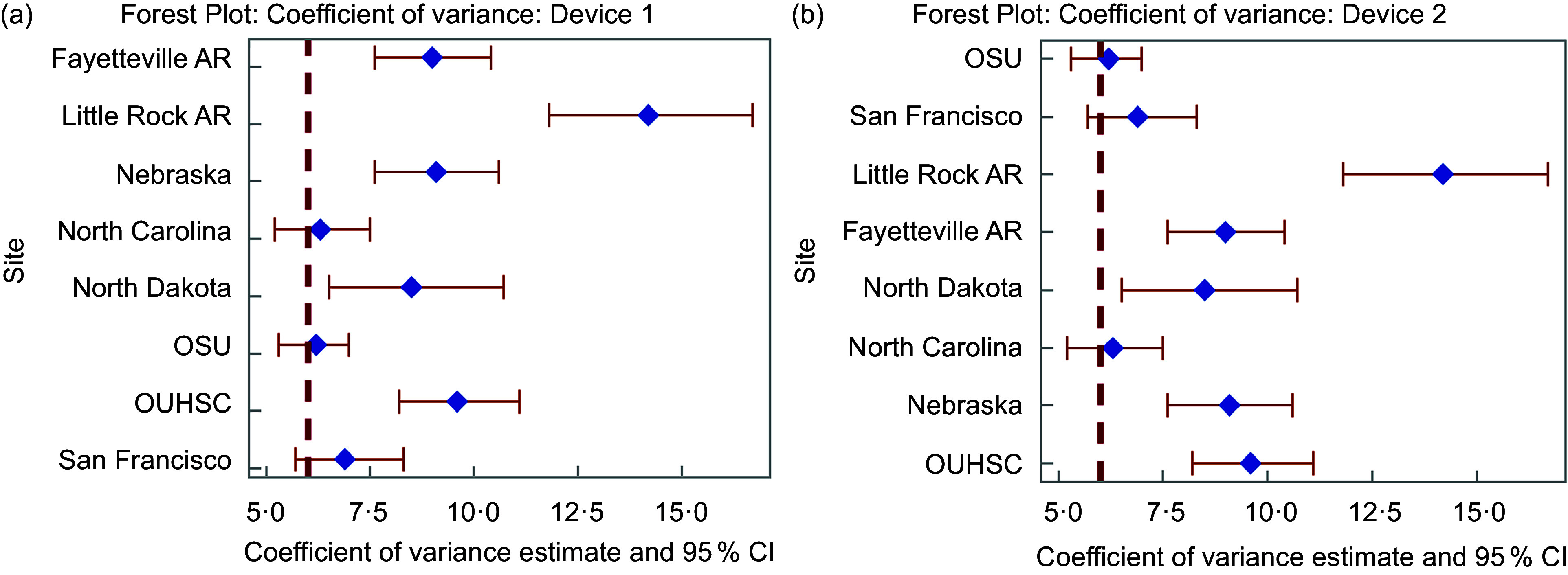
(a) Forest plot demonstrating CV and 95 % CI for Veggie Meter® device 1 at each site. Bold, dashed line indicated the hypothesised CV. (b) Forest plot demonstrating CV and 95 % CI for Veggie Meter® Device 2 at each site. Bold, dashed line indicated the hypothesised CV. AR, Arkansas; OSU, Oklahoma State University; OUHSC, University of Oklahoma Health Sciences Center.

Intra-device ICC can be found in Table 2. Intra-device ICC ranged from 0·77 to 0·99, with an average of 0·89 indicating good to excellent agreement within SCS on the same Veggie Meter® device. Table 3 displays each device’s five individual and average of five SCS. CV was hypothesised to be 6 % based on previous literature^(18,20)^ and is a measure of the dispersion of the five scores per device within an individual participant. CV for devices 1 and 2 can be found in Figure 1(a) and (b) and Table 2. The CV ranges from 6·2 % to 14·2 %, with an average of 8·8 %. A majority (63 %; *n* 10) of the Veggie Meter® devices had significantly higher CV from the hypothesised 6 %. Further, we observed no patterns of association between equipment variability reported in online supplementary material, Supplemental Table 1 and measures of intra-device reliability.

**Table 2:** Intra- and inter-device reliability output across 8 sites

	OUHSC (*n* 35)		UNL (*n* 35)		ECU/NC State (*n* 35)		UAMS – Fayetteville (*n* 33)		UAMS – Little Rock (*n* 37)		OSU (*n* 37)		GFNHRC (*n* 35)		SFDPH (*n* 35)	
Intra-device																
Device 1																
Intra ICC (95 % CI)	0·92	0·87, 0·95	0·88	0·80, 0·93	0·91	0·85, 0·95	0·82	0·73, 0·89	0·79	0·69, 0·87	0·95	0·92, 0·97	0·93	0·88, 0·96	0·94	0·90, 0·96
Intra CV (95 % CI)	9·6 %	8·2, 11·1	9·1 %	7·6, 10·6	6·3 %	5·2, 7·5	9·0 %	7·6, 10·4	14·2 %	11·8, 16·7	6·2 %	5·3, 7·0	8·5 %	6·5, 10·7	6·9 %	5·7, 8·3
Device 2																
Intra ICC (95 % CI)	0·91	0·86, 0·95	0·88	0·81, 0·93	0·94	0·90, 0·97	0·77	0·66, 0·86	0·78	0·68, 0·87	0·99	0·89, 0·96	0·93	0·89, 0·96	0·95	0·93, 0·97
Intra CV (95 % CI)	8·6 %	7·7, 9·6	9·1 %	7·7, 10·4	6·3 %	5·3, 7·4	10·3 %	8·9, 12·0	14·0 %	11·7, 16·5	7·2 %	6·0, 8·4	8·1 %	6·4, 10·1	7·6 %	6·4, 8·8
Inter-device																
Inter ICC (95 % CI)	0·88	0·84, 0·91	0·86	0·81, 0·90	0·58	0·30, 0·73	0·75	0·68, 0·81	0·65	0·55, 0·73	0·94	0·92, 0·96	0·91	0·89, 0·94	0·89	0·57, 0·96
Abs Rel Diff (95 % CI)	13·5 %	11·9, 15·1	10·9 %	8·9, 13·1	19·3 %	14·3, 24·6	12·7 %	10·5, 15·1	22·0 %	17·9, 26·5	7·5 %	6·6, 8·5	11·8 %	8·7, 15·5	13·4 %	10·9, 16·0

ICC, intra-class correlation coefficients; OUHSC, Oklahoma Health Sciences in Oklahoma City; UNL, University of Nebraska in Lincoln, Nebraska; ECU, East Carolina University; NC, North Carolina; UAMS, University of Arkansas for Medical Sciences; OSU, Oklahoma State University; SFDPH, San Francisco Department of Public Health.

**Table 3:** Individual Veggie Meter® skin carotenoid scores and average for each site

	Veggie Meter® 1		Veggie Meter® 2	
	Mean	sd	Mean	sd
OUHSC				
Scan 1	293·2	95·7	301·1	95·8
Scan 2	291·9	93·3	300·9	87·0
Scan 3	291·2	91·0	299·5	86·9
Scan 4	292·9	95·7	289·0	87·7
Scan 5	287·6	90·3	292·4	90·0
Average	291·3	90·0	296·6	86·3
UNL				
Scan 1	301·5	75·3	273·9	77·7
Scan 2	278·0	73·1	292·5	74·6
Scan 3	298·6	82·0	281·3	79·2
Scan 4	276·8	83·8	287·0	79·6
Scan 5	293·9	73·7	269·1	66·4
Average	289·7	74·3	280·8	72·4
ECU/NC State				
Scan 1	280·6	58·3	243·2	70·4
Scan 2	271·7	59·6	245·7	65·7
Scan 3	281·2	64·2	241·4	66·2
Scan 4	270·8	65·3	236·1	65·1
Scan 5	268·0	66·4	238·7	68·2
Average	274·5	60·6	241·0	65·6
UAMS – Fayetteville				
Scan 1	290·6	67·8	301·4	66·9
Scan 2	300·9	68·3	304·4	73·8
Scan 3	292·4	60·5	298·2	64·4
Scan 4	296·7	62·0	291·3	71·9
Scan 5	285·5	70·3	298·3	70·2
Average	293·2	61·0	298·7	62·8
UAMS – Little Rock				
Scan 1	217·4	80·0	245·8	79·1
Scan 2	228·7	83·2	232·2	80·6
Scan 3	232·1	82·4	244·1	89·9
Scan 4	225·8	77·2	245·4	89·4
Scan 5	226·9	81·5	233·8	76·0
Average	262·2	73·6	240·3	75·7
OSU				
Scan 1	302·3	85·9	294·9	88·8
Scan 2	305·8	90·4	310·2	92·1
Scan 3	316·7	93·8	310·2	87·4
Scan 4	312·7	87·7	311·4	88·0
Scan 5	311·3	87·6	306·4	88·0
Average	309·8	87·3	306·6	86·5
GFNHRC				
Scan 1	275·1	82·2	282·3	83·7
Scan 2	274·6	82·7	277·5	87·4
Scan 3	274·8	84·6	273·0	83·9
Scan 4	280·3	89·8	274·1	94·5
Scan 5	278·3	92·8	278·3	88·4
Average	276·6	83·9	277·1	85·3
SFDPH				
Scan 1	323·2	96·8	288·6	103·0
Scan 2	336·0	99·4	296·7	101·9
Scan 3	323·2	94·4	296·7	104·0
Scan 4	320·9	95·8	294·7	100·9
Scan 5	324·9	95·8	298·5	99·0
Average	325·6	94·1	295·0	99·9

OUHSC, Oklahoma Health Sciences in Oklahoma City; UNL, University of Nebraska in Lincoln, Nebraska; ECU, East Carolina University; NC, North Carolina; UAMS, University of Arkansas for Medical Sciences; OSU, Oklahoma State University; GFHNRC, Grand Forks Human Nutrition Research Center in Grand Forks, North Dakota; SFDPH, San Francisco Department of Public Health.

**Figure 2:**
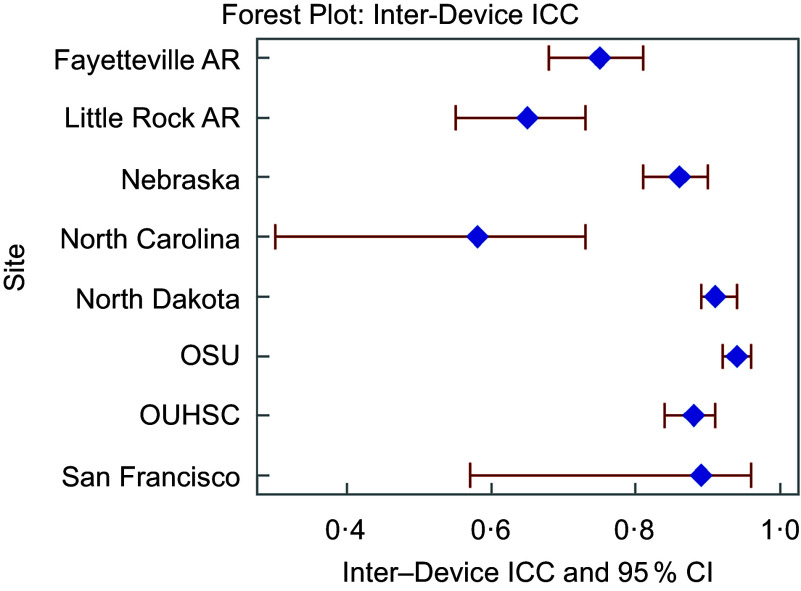
Inter-device intra class correlation coefficients and 95 % CI for each site. AR, Arkansas; OSU, Oklahoma State University; OUHSC, University of Oklahoma Health Sciences Center.

**Figure 3:**
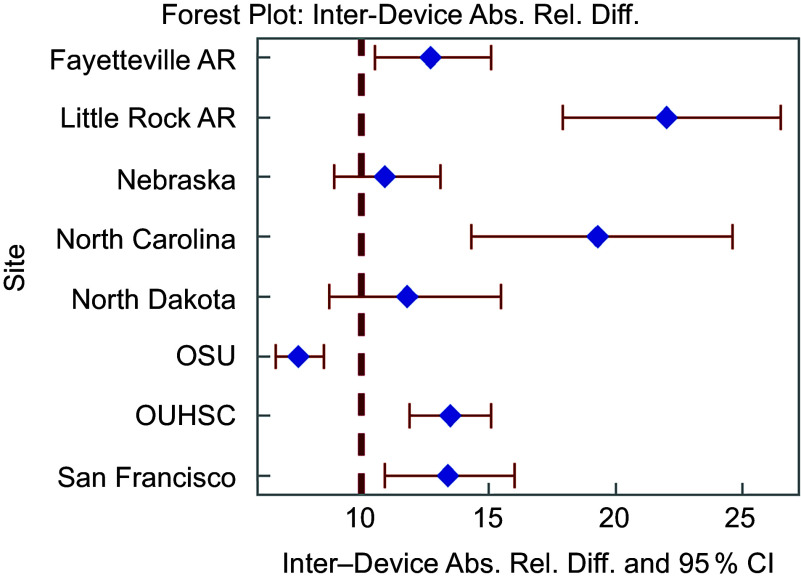
Inter-device absolute relative difference and 95 % CI for each site. AR, Arkansas; OSU, Oklahoma State University; OUHSC, University of Oklahoma Health Sciences Center. Bold, dashed line indicated the hypothesized absolute relative difference.

**Figure 4:**
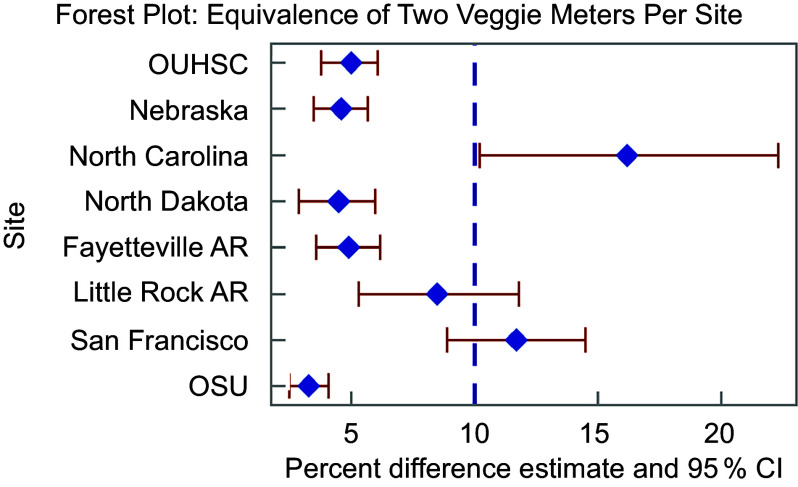
Equivalence of both Veggie Meter® devices and 95 % CI for each site. Bold, dashed line indicated the hypothesized equivalence. AR, Arkansas; OSU, Oklahoma State University; OUHSC, University of Oklahoma Health Sciences Center.

Inter-device ICC across both devices at each site can be found in Figure 2 and Table 2. The range of ICC indicating degree of agreement between Veggie Meter® devices for a single individual varies between 0·58 and 0·94, indicating moderate to excellent agreement. ARD was hypothesised to be 10 % based on previous research^(20)^. Figure 3 and Table 2 show the ARD across devices. The ARD ranged from 7·5 % to 22·0 %, with an average of 13·9 %. A majority (*n* 5) of sites had an ARD significantly higher than the hypothesised 10 % ranging from 12·7 % to 22 %; one site had a significantly lower ARD (7·5 %) and the ARD for two sites (25 %) did not significantly differ from the hypothesised 10 %. Equivalence at each threshold including 5 % CI is in Figure 4. Five sites (63 % of all sites) demonstrated equivalence below the hypothesised 10 % threshold indicating SCS from one device would be expected to be within 10 % of the other device. Further, we observed no patterns of association between equipment variability reported in online supplementary material, Supplemental Table 1 and measures of inter-device reliability.

## Discussion

The primary findings of this study indicate that *within* Veggie Meter® devices, there is good to excellent agreement across SCS. However, even with high correlations within each device, the variation within an individual was larger than the predicted 6 % in the majority (63 %) of sites ranging from 6 % to 14 %, with an average of 8·8 %. These values are slightly higher compared with the degree of variation between scores using resonance Raman spectroscopy (5·7 %) reported in another study^(27)^, although the study did not report variation for RS to compare. Education, behavioural and community environmental interventions have reported intervention changes in Veggie Meter® assessed SCS ranging from 3 % to 58 %^(1–4,13,19,28–36)^, while supplementation studies generally have larger effects (range 5–58 %)^(13,19,28–31)^. Our results demonstrate the need to interpret changes in Veggie Meter®-assessed SCS findings with consideration for the margin of difference.

Studies examining resonance Raman spectroscopy variability *between* devices report CV ranges of 0·5–14·6 %, with an average of 5·1 %^(37,38)^. This is smaller than the average CV of 8·8 % observed in our study. There was moderate to high agreement across different Veggie Meter® SCS for a single individual. While the majority (63 %) of sites demonstrated equivalence below 10 %, the ARD, sites demonstrated variability ranging from 7·5 % to 22·0 % with an average of 13·9 %. This degree of inter-device variability necessitates caution when using multiple devices on a single participant over time. While the maximum difference between devices was 22 %, the average difference across all sites was approximately 14 %.

### Practical implications

Several practical implications have emerged from this study. The highest CV was 14 %, conservatively indicating that actual changes observed may need to exceed 14 % to be clearly attributed to the intervention and outside the device margin of difference within an individual. Agreement across devices was moderate to high, with the range of difference being 12–22 %. Thus, to reduce artifacts and noise in the data, it is recommended that the same Veggie Meter® be used to measure an individual over time. If tracking devices is not feasible in the context or multiple devices are used across a study, ARD across devices can be calculated per site and reported to assure that change observed is attributable to the intervention effects and larger than the margin of difference between devices. The variance between scores should also be considered when comparing Veggie Meter® scores across papers and studies.

Manufacturer (Longevity Link, LLC) recommendations state that field ‘recalibration’ using the light and dark calibration sticks should be conducted every hour of continual use^(22)^. One site (UNL) used light and dark calibration sticks every 30 min. One could hypothesise that perhaps that due to the more frequent use of the recalibration process, the degree of variability within and between participants would be decreased. However, this was not the case and the degree of variability within and between at UNL was equal to or greater than other sites. Further, the optimal frequency or regularity of service or calibration at Longevity Link headquarters is unknown. Specifically, it is currently unknown what impact servicing or re-calibration when devices are returned to Longevity Link has on the variability of SCS. Researchers may wish to avoid returning a device for service in the middle of a study or document such occurrences if they are unavoidable. In efforts to promote transparency and standardise reporting of SCS, dissemination efforts could include age of the device, equipment use patterns and date of last calibration.

### Research opportunities

Based on our findings, the calculation of a correction factor for more accurate translation across multiple devices may be beneficial. Furthermore, while we found a 14 % margin of difference, an acceptable range of difference should also be defined. The FDA has determined that all self-monitoring blood glucose test kits may have a maximum of ±15 % error rate for about 95 % of the measurements, including the kits which use spectroscopy^(39)^. Skin roughness and oxidative exposure to the sun are both reported to impact skin carotenoids^(40,41)^ and developing greater understanding of these impacts and how to navigate them in controlled research and public health settings would be needed. Additional needs for research in a variety of populations include accuracy, validity, reliability and translation of Veggie Meter®-assessed SCS.

Treatment of spurious values was not addressed in the recommended standard Veggie Meter® research protocol^(22)^. While the majority of the sites in this study recorded each value without any repositioning, cleaning equipment or recalibration, two sites had a more conservative approach to spurious values (UNL and GFNHRC). These sites rescanned the finger if the score was >10 % (UNL) or 100 points (GFNHRC) from the previous scan and discarded spurious values. UNL went so far as to recalibrate the Veggie Meter® then reposition the finger. With this more conservative approach, one would hypothesise that the intra- and inter-device reliability would be higher. However, this was not the case, as the CV and ADR for both sites were in the middle of the range across sites. While it seems prudent to reposition the finger if value appears spurious, the appropriate protocol for that process needs to be determined. It does not appear that discarding spurious values at these sites impacted the reliability of the SCS.

The development of field calibration phantoms or sticks that can be scanned with a known carotenoid score would be a critically important development to ensure Veggie Meter® devices are indeed accurately recording in the field. If multiple Veggie Meters® SCS are not regularly compared, accuracy issues could be overlooked. The ability for users to determine the accuracy of a device in the field, without another Veggie Meter®, is a critical next step to ensuring the integrity of Veggie Meter® utilisation in research projects.

These findings should be considered in light of the strengths and limitations of this study. Strengths of our study design include numerous sites across the USA representing different geographic regions, climates, sun exposure and month of the year. However, this is also a limitation since the Veggie Meter® devices used at each site were not the same and purchased at different times, had been serviced at different times and used in a variety of settings. This variability may have introduced bias or error into the data collected. The demographic and racial representation at each of these sites varied, which may contribute to the intra- and inter-device reliability at each site. While previous research demonstrates no differences in SCS by race^(18)^, more research is needed to understand these relationships more clearly. Another strength of our study was the diversity of ethnicities, age, skin reactivity categories and weight status. All efforts were taken to minimise systematic bias introduced by data collection procedures such as rigorous timing of scans, cleaning of participant fingers, counter-balancing and a series of five scans. Additional limitations include minor deviations on site procedures. Site investigators met virtually to develop the protocol and throughout data collection. A single IRB was considered across sites and not pursued given the cross-sectional nature of this project and varying institutional review board protocol requirements.

### Conclusion

Veggie Meters® is an increasingly popular method of non-invasive dietary assessment in community populations and to evaluate intervention effectiveness. Our study demonstrates the intra-device and inter-device reliability to be moderate to high, as per ICC. We offer guidance that the observed margin of difference within a device was up to 14 %, with an average of 9 %. We offer guidance that the observed margin of difference between devices was up to 22 % with an average of 14 % between devices. Practical implications support previous work^(22)^ and include utilisation of the same equipment for a participant across an intervention timeframe and the utilisation of multiple finger scans (i.e. triplicate mode or average multiple individual scans). More research is needed to refine the accurate use and ability to translate Veggie Meter (R)-assessed SCS in public health nutrition research studies.

## Supporting information

Sisson et al. supplementary material Sisson et al. supplementary material

## Supplementary material

For supplementary material accompanying this paper visit https://doi.org/10.1017/S136898002510116X

## References

[1] Akingbule O , Teran-Garcia M , Okoye S et al. (2023) Impact of a nutrition education and physical activity intervention on fruits and vegetable intake of Nigerian immigrants. J Nutr Educ Behav 55, 79–80. doi: 10.1016/j.jneb.2023.05.173.

[2] Di Noia J , Monica D , Sikorskii A et al. (2021) Pilot study of a farm-to-special supplemental nutrition program for Women, Infants, and Children (WIC) intervention promoting vegetable consumption. J Acad Nutr Diet 121, 2035–2045. doi: 10.1016/j.jand.2020.12.020. 33487590 PMC8295404

[3] Laviolette C , Johnson CM , Butler JL et al. (2023) Nutrition effects of a family-centered health promotion program for Mexican-Heritage children in the lower Rio Grande Valley of Texas. Nutrients 15, 1600. doi: 10.3390/nu15071600. 37049438 PMC10097021

[4] Obana A , Asaoka R , Miura A et al. (2022) Improving skin carotenoid levels in young students through brief dietary education using the Veggie Meter. Antioxidants 11, 1570. doi: 10.3390/antiox11081570. 36009289 PMC9405129

[5] Bakirci-Taylor AL , Reed DB , McCool B et al. (2019) mHealth improved fruit and vegetable accessibility and intake in young children. J Nutr Educ Behav 51, 556–566. doi: 10.1016/j.jneb.2018.11.008. 30638880

[6] Caparello G , Ceraudo F , Meringolo F et al. (2024) Eating habits and carotenoid skin content among children based on their attendance at the school meals: a cross-sectional pilot study. J Clin Transl Endocrinol 38, 100378. doi: 10.1016/j.jcte.2024.100378. 39659433 PMC11629320

[7] Hill CM , Paschall MJ , O’Brien DM et al. (2021) Characterizing vegetable and fruit intake in a remote Alaska native community using reflection spectroscopy and 24-hour recalls. J Nutr Educ Behav 53, 712–718. doi: 10.1016/j.jneb.2021.02.002. 33715972 PMC8783602

[8] Whiteside-Mansell L , Swindle TM & Davenport K (2019) Evaluation of ‘Together, We Inspire Smart Eating’ (WISE) nutrition intervention for young children: assessment of fruit and vegetable consumption with parent reports and measurements of skin carotenoids as biomarkers. *J Hunger Environ Nutr*. Published online: 19 August 2019. doi: 10.1080/19320248.2019.1652127. PMC7597824 33133327

[9] Ermakov IV , Ermakova M , Sharifzadeh M et al. (2018) Optical assessment of skin carotenoid status as a biomarker of vegetable and fruit intake. Arch Biochem Biophys 646, 46–54. doi: 10.1016/j.abb.2018.03.033. 29601824 PMC6370053

[10] Jahns L , Johnson LK , Conrad Z et al. (2019) Concurrent validity of skin carotenoid status as a concentration biomarker of vegetable and fruit intake compared to multiple 24-h recalls and plasma carotenoid concentrations across one year: a cohort study. Nutr J 18, 78. doi: 10.1186/s12937-019-0500-0. 31752882 PMC6873686

[11] Jilcott Pitts SB , Moran NE , Wu Q et al. (2022) Pressure-mediated reflection spectroscopy criterion validity as a biomarker of fruit and vegetable intake: a 2-site cross-sectional study of 4 racial or ethnic groups. J Nutr 152, 107–116. doi: 10.1093/jn/nxab349. 34562088 PMC8754514

[12] Casperson SL , Roemmich JN , Larson KJ et al. (2023) Sensitivity of pressure-mediated reflection spectroscopy to detect changes in skin carotenoids in adults without obesity in responses to increased carotenoid intake: a randomized controlled trial. J Nutr 153, 588–597. doi: 10.1016/j.tjnut.2023.01.002. 36894250

[13] Jilcott Pitts S , Moran NE , Laska MN et al. (2023) Reflection spectroscopy-assessed skin carotenoids are sensitive to change in carotenoid intake in a 6-week randomized controlled feeding trial in a racially/ethnically diverse sample. J Nutr 153, 1133–1142. doi: 10.1016/j.tjnut.2023.02.017. 36804322 PMC10356992

[14] Rush E , Jalili-Moghaddam S , Diep T et al. (2019) Who is eating their veggies. Proceedings 37, 14. doi: 10.3390/proceedings2019037014.

[15] Martinelli S , Acciai F , Tasevska N et al. (2021) Using the Veggie Meter in elementary schools to objectively measure fruit and vegetable intake: a pilot study. Methods Protoc 4, 33. doi: 10.3390/mps4020033. 34066275 PMC8162554

[16] Varghese V , Cepni AB , Change J et al. (2023) Skin carotenoids measures by reflection spectroscopy correlate wtih dietary carotenoid intake in racially and ethnically diverse US toddlers from Houston, Texas. J Acad Nutr Diet 124, 628–635.e1. doi: 10.1016/j.jand.2023.10.015. 39491166

[17] Di Noia J & Gellermann W (2021) Use of the spectroscopy-based Veggie Meter((R)) to objectively assess fruit and vegetable intake in low-income adults. Nutrients 13, 2270. doi: 10.3390/nu13072270. 34209048 PMC8308249

[18] Jilcott Pitts SB , Jahns L , Wu Q et al. (2018) A non-invasive assessment of skin carotenoid status through reflection spectroscopy is a feasible, reliable and potentially valid measure of fruit and vegetable consumption in a diverse community sample. Public Health Nutr 21, 1664–1670. doi: 10.1017/S136898001700430X. 29455692 PMC6200334

[19] Casperson SL , Scheett A , Palmer DG et al. (2023) Biochemical validation of a self-administered carotenoid intake screener to assess carotenoid intake in nonobese adults. Curr Dev Nutr 7, 100024. doi: 10.1016/j.cdnut.2022.100024. 37180085 PMC10111597

[20] Obana A , Asaoka R , Takayanagi Y et al. (2023) Inter-device concordance of Veggie Meter-a reflection spectroscopy to measure skin carotenoids. J Biophotonics 16, e202300071. doi: 10.1002/jbio.202300071. 37072378

[21] Fitzpatrick TB (1988) The validity and practicality of sun-reactive skin types I through VI. Arch Dermatol 124, 869–871. doi: 10.1001/archderm.124.6.869. 3377516

[22] Radtke MD , Poe M , Stookey J et al. (2021) Recommendations for the use of the Veggie Meter(R) for spectroscopy-based skin carotenoid measurements in the research setting. Curr Dev Nutr 5, nzab104. doi: 10.1093/cdn/nzab104. 34476333 PMC8405124

[23] Ermakov IV & Gellermann W (2012) Dermal carotenoid measurements via pressure mediated reflection spectroscopy. J Biophotonics 5, 559–570. doi: 10.1002/jbio.201100122. 22331637

[24] Roberts WE (2009) Skin type classification systems old and new. Dermatol Clin 27, 529–533, viii. doi: 10.1016/j.det.2009.08.006. 19850202

[25] Koo TK & Li MY (2016) A guideline of selecting and reporting intraclass correlation coefficients for reliability research. J Chiropr Med 15, 155–163. doi: 10.1016/j.jcm.2016.02.012. 27330520 PMC4913118

[26] Liljequist D , Elfving B & Skavberg Roaldsen K (2019) Intraclass correlation – a discussion and demonstration of basic features. PloS One 14, e0219854. doi: 10.1371/journal.pone.0219854. 31329615 PMC6645485

[27] Hwang JE , Park JY , Jung MH et al. (2023) Evaluation of a commercial device based on reflection spectroscopy as an alternative to resonance Raman spectroscopy in measuring skin carotenoid levels: randomized controlled trial. Sens 23, 7654. doi: 10.3390/s23177654. PMC10490775 37688110

[28] Yamane Y , Mochiji M , Ichioka S et al. (2022) Effects of water chestnut (Tarpa bispinosa Roxb.) extract/lutein on fingertip-measured advanced glycation endproduct/carotenoid levels. Free Radic Res 56, 282–289. doi: 10.1080/10715762.2022.2085098. 35687659

[29] Obana A , Gohto Y , Nakazawa R et al. (2020) Effect of an antioxidant supplement containing high dose lutein, zeaxanthin on macular pigment, skin carotenoid levels. Sci Rep 10, 10262. doi: 10.1038/s41598-020-66962-2. PMC7314813 32581313

[30] Martell SG , Kim J , Cannavale CN et al. (2023) Randomized, placebo-controlled, single-blind study of lutein supplementation on carotenoid status and cognition in persons with multiple sclerosis. J Nutr 153, 2298–2311. doi: 10.1016/j.tjnut.2023.06.027. 37364683 PMC10447884

[31] Amoah I , Cairncross C & Rush E (2023) Vegetable-enriched bread: pilot and feasibility study of measurement of changes in skin carotenoid concentrations by reflection spectroscopy as a biomarker of vegetable intake. Food Sci Nutr 11, 3376–3384. doi: 10.1002/fsn3.3327. 37324838 PMC10261728

[32] Jilcott Pitts SB , Wu Q , Truesdale KP et al. (2018) One-year follow-up examination of the impact of the north carolina healthy food small retailer program on healthy food availability, purchases, and consumption. Int J Environ Res Public Health 15, 2681. doi: 10.3390/ijerph15122681. 30487427 PMC6313329

[33] Biddle MJ , Kang JJ , Brewer D et al. (2021) Reducing cardiovascular risk among participants in a community supported agriculture program. Circulation 144, A10560. doi: 10.1161/circ.144.suppl_1.10560.

[34] Khalil M & Moore C (2022) The impact of an after-school Garden Enhanced Education (GENE) on skin carotenoids as a measure of vegetable consumption on diverse, low-income children. Cur Dev Nutr 6, 60. doi: 10.1093/cdn/nzac049.006.

[35] Bayles J , Peterson AD , Jilcott Pitts S et al. (2021) Food-based Science, Technology, Engineering, Arts, and Mathematics (STEAM) learning activities may reduce decline in preschoolers’ skin carotenoid status. J Nutr Educ Behav 53, 343–351. doi: 10.1016/j.jneb.2020.10.017. 33349594 PMC8044028

[36] Ling J , Suriyawong W , Robbins LB et al. (2024) FirstStep2Health: a cluster randomised trial to promote healthy behaviours and prevent obesity amongst low-income preschoolers. Pediatr Obes 19, e13122. doi: 10.1111/ijpo.13122. 38622494 PMC11156553

[37] Ermakov IV & Gellermann W (2010) Validation model for Raman based skin carotenoid detection. Arch Biochem Biophys 504, 40–49. doi: 10.1016/j.abb.2010.07.023. 20678465

[38] Bernstein PS , Zhao DY , Wintch SW et al. (2002) Resonance Raman measurement of macular carotenoids in normal subjects and in age-related macular degeneration patients. Ophthalmology 109, 1780–1787. doi: 10.1016/s0161-6420(02)01173-9. 12359594 PMC3079575

[39] Food and Drug Administration (2020) Blood Glucose Monitoring Test Systems for Prescription Point-Of-Care Use; Guidance for Industry and Food and Drug Administration Staff. Rockville, MD: Food and Drug Administration.

[40] Longevity Link Corporation (2016) The Veggie Meter. Salt Lake City, UT: Longevity Link Corporation. http://www.longevitylinkcorporation.com/products.html (accessed october 2025).

[41] Madore MP , Hwang JE , Park JY et al. (2023) A narrative review of factors associated with skin carotenoid levels. Nutrients 15, 2156. doi: 10.3390/nu15092156. 37432294 PMC10180675

