# Supplementary material for: Examining intra- and inter-device reliability of pressure-mediated reflection spectroscopy in a multi-state sample of healthy adults
Source: Public Health Nutr. 2025 Sep 25;28(1):e169. doi: 10.1017/S136898002510116X (PMC12722093; doi:10.1017/S136898002510116X)
Supplement: Sisson et al. supplementary material [file S136898002510116Xsup001.docx]

Supplementary Table 1. Veggie Meter® Equipment and Protocol Variations Encountered

|  | OUHSC | UNL | ECU/NC State | UAMS- Fayetteville | UAMS - Little Rock | OSU | GFNHRC | SFDPH |
| --- | --- | --- | --- | --- | --- | --- | --- | --- |
| Year purchased | Oct 2020 | 2021 | 2018 | 2021 | 2019 (1)  2021 (2) | 2022 | 2017 (1)  2019 (2) | 2020 (1)  2020 (2) |
| Last date of equipment service | June 2021 (both) | n/a | July/July 2022 | n/a | August 2023 | n/a | 06/09/2022 (both) | n/a  (first use  out of box) |
| Model numbers | VM200166  VM200167 | VM200196  VM200195 | 318W0017  816W0096 | VM200186 VM200188 | VM2000198  VM200212 | VM200240VM200239 | 716W0088 (1)  617W0039 (2) | VM200116 (1) VM200120 (2) |
| White reference range | 12,000-15,000 | 12,000-15,500 | 42,000-56,000 | 12,000-15,000 | 12,000-15,500 (1,2) | 47,000-61,000 (1)  47,000-61,000  (2) | 45,000 – 54,000 (1)  49,000 – 56,000 (2) | 12,000-15,000 (1)  12,000-15,000 (2) |
| Dark reference range | 1,580±1,000 | 1,570±1,000 | 9,500-10,500 | 1,580±1000 | 1,570±1,000 (1)  1,560 ±1,000 (2) | “~170 ± 200” (1)  “~170 ± 200” (2) | 9,142±1,000 (1)  8,377±1,000 (2) | 1,600±1,000 (1)  1,600±1,000 (2) |
| Warm up instructions | No warm up needed if constant temperature. 15 minutes otherwise | No warm up needed | Warm up a minimum of 5 minutes | No warm up needed if constant temperature. 15 minutes otherwise | No warm up needed if constant temperature. 15 minutes otherwise | No warm up needed if constant temperature. 15 minutes otherwise | Warm up a minimum of 5 minutes | 15 minutes to warm up |
| Number of technicians collecting data across the study | 1 | 2 | 1 | 7 | 6 |  | 2 | 4 |
| Scheduling | Single participant | Single participant | Single participant | Alternating participants | Alternating participants | Single participant | Single participant | Alternating participants |
| Calibration | Prior to scans and hourly thereafter | Prior to scans and every half hour thereafter | Prior to scans and hourly thereafter | Prior to scans and hourly thereafter | Prior to scans and hourly thereafter | Prior to scans and hourly thereafter | Prior to scans and hourly thereafter | Prior to scans, then every hour |
| Spurious values | Recorded each value without repositioning or recalibration | If value was greater than 10% different from previous scan, the device was recalibrated and participant rescanned. Spurious value was discarded | Recorded each value without repositioning or recalibration | Recorded each value without repositioning or recalibration | Recorded each value without repositioning or recalibration | Recorded each value without repositioning or recalibration | If value was greater than 100 points different from previous scan, finger was repositioned and rescanned. Spurious value discarded | Recorded each value without repositioning or recalibration |
| Survey distribution | Completed online by participant | Completed online by participant | Completed online by participant | Completed online by participant | Completed online (on site via Ipad) by participant | Completed on paper and entered into online database | Completed on paper and entered into online database | Completed on paper and entered into online database |
| Other exclusions | Participants who had eaten Cheetos, Doritos, Takis in prior 3 days or used self-tanner within 2 weeks | No other exclusions | No other exclusions | Participants who had eaten Cheetos, Doritos, Takis in prior 3 days or used self-tanner within 2 weeks | No other exclusions | Participants who had eaten Cheetos, Doritos, Takis in prior 3 days or used self-tanner within 2 weeks | Used self-tanners within 2 weeks | Acute illness  Self-reported a chronic health condition |

Supplementary Table 2. Description of Anthropometric Equipment Used at Each Site

| Site | Stadiometer | Scale | Source |
| --- | --- | --- | --- |
| OUHSC | Seca Stadiometer | Seca 770 Scale | Seca, North America, Chino, Ca |
| UNL | Seca Stadiometer | Seca 813 | Seca, North America, Chino, Ca |
| ECU/NC State | Seca 213 Portable Stadiometer | TANITA DC-430U | Seca, North America, Chino, CA  TANITA, North America, Arlington Heights, IL |
| UAMS-Fayetteville | Not collected | Not collected | n/a |
| UAMS-Little Rock | Self-reported | Self-reported | n/a |
| OSU | Seca Stadiometer | Seca mBCA 514 | Seca, North America, Chino, Ca |
| GFHNRC | Easy-Glide Bearing Stadiometer | Ultegra Series Digital Health Scale | Stadiometer: Perspective Enterprises, Portage, MI  Scale: Fairbanks Scales, Overland Park, KS |
| SFDPH | Self-reported | Self-reported | n/a |
